# Mental Distress during the Coronavirus Pandemic in Israel: Who Are the Most Vulnerable?

**DOI:** 10.3390/ijerph19010124

**Published:** 2021-12-23

**Authors:** Tehila Refaeli, Michal Krumer-Nevo

**Affiliations:** The Charlotte Jack Spitzer Department of Social Work, Ben-Gurion University of the Negev, Beer-Sheva 84105, Israel; kmichal@bgu.ac.il

**Keywords:** mental distress, mental health, COVID-19, unemployment, economic effects, socioeconomic status, social support

## Abstract

Based on Pearlin’s stress process model and the social inequality approach to health, this study used a social lens to explore the role of socioeconomic inequities in mental distress during the COVID-19 pandemic in Israel. Specifically, we examined people’s pre-pandemic sociodemographic characteristics and economic situation, and the economic effects of the pandemic itself on mental distress. A real-time survey was conducted in May 2020 among 273 adults (ages 20–68), and hierarchical linear models were employed. Findings indicated that groups vulnerable to mental distress in routine times (e.g., women, people with economic difficulties) showed the same pattern during the pandemic. Not only was unemployment related to mental distress, so too was a reduction in work hours. The pandemic’s economic effects (e.g., needing to take out loans, having a worsening financial situation) were also associated with increased mental distress. This study is one of very few studies to explore a wide range of socioeconomic factors and their association with mental distress during the current crisis. The findings call for broader interventions to alleviate the economic distress caused by the pandemic to promote mental health, especially for groups that were vulnerable before the crisis and those most affected economically following the pandemic.

## 1. Introduction

The literature concerning disparities in terms of the physical health effects of COVID-19 has been growing rapidly in the months following the pandemic’s outbreak. At this point, it is clear that people living in poverty or with a low socioeconomic status (SES), minorities, and people with preexisting medical conditions are at a higher risk of being infected by the virus [1,2,3]. Less attention has been given to the factors that make people vulnerable to the mental health effects of the pandemic, and to the contribution of various marginal social positions to such mental health effects.

The pandemic has various effects beyond the well-documented physical health effects worldwide. With time, the economic damage wrought by the pandemic in the global and individual sphere are becoming more clear [4,5,6,7], specifically among low SES groups [8]. One main reason for this economic devastation is the high rates of unemployment, furlough, and decrease in work hours around the globe in the wake of the pandemic [9,10]. That said, the relationship between these economic effects and people’s mental health is less explored. Moreover, there is a dearth of knowledge regarding the role played by factors associated with people’s situations prior to the pandemic vs. factors associated with the harm caused by the pandemic. To narrow this gap, the current study aimed to explore which factors predicted mental distress during the pandemic, including sociodemographic factors such as SES prior to the COVID-19 outbreak, and the various economic effects wrought by this once-in-a-century global health crisis.

### 1.1. Mental Distress and Associated Factors

Mental health is an integral part of the definition of overall health by the World Health Organization (WHO) [11], as it “enables people to realize their potential, cope with stressors in life, work productively and contribute to their communities” (WHO, 2013, p.5). In this context, mental distress is defined as a collection of mental health problems and mental health disorders that do not fall into standard diagnostic criteria and are characterized by symptoms such as sleeping problems, irritability, difficulty in concentrating, etc. [12,13]. Mental distress is strongly connected with impaired physical health and mortality [14,15].

Various factors have been identified as having an association with mental distress. Contextual factors related to SES indicators, such as education and employment, are related to mental distress [13,16,17,18]. There is also a vast knowledge of the direct effect of social capital and social support on mental health outcomes [19,20,21,22]. In addition, personal resources, such as self-esteem, optimism, and mastery are important in predicting mental health or distress [18,23,24].

Studies have found that COVID-19–an external stressor–poses a threat to the mental health of various populations around the globe [23,25,26,27,28]. For example, an APA (American Psychological Association) poll indicated a sharp increase in the level of people reporting anxiety in the U.S. during the coronavirus pandemic (62%) compared to the levels of the previous three years (32–39%) [25]. Given that COVID-19 poses a universal threat to people’s mental health globally, it is important to explore which factors specifically contribute to mental distress amidst this health and economic crisis on people across various social positions.

### 1.2. Theoretical Background

The theoretical framework that was adopted for this study is the stress process model [29]. This theory refers to the effects of stress on mental health in general, and suggests that stressors or strains, such as a low SES, living in an unsafe neighborhood, and having a serious illness are stressful life conditions [30]. This theory also highlights the idea that psychological resources (coping, self-esteem, mastery) and social resources (social support) can decrease the effects of stressors on mental health [30]. The stress process model is especially relevant to the current study, as Pearlin [30] claimed that the effect of stressful life events, such as the coronavirus pandemic, on the life of lower-status groups (for instance, racial/ethnic minorities, immigrants, and the economically disadvantaged) should be explored separately from their effect on their more advantaged counterparts. These disadvantaged groups can experience greater and more powerful stressors in the aftermath of the same events [31,32].

Additionally, Pearlin [30] indicated that significant stressors rarely occur in a vacuum. Namely, one stressor is very likely to trigger other stressors. For example, unemployment would likely lead to economic strain which would likely, in a snowball-like fashion, lead to other disparities. Accordingly, in the current study we assumed that although the stress caused by the pandemic increased mental distress for most people, it had a greater detrimental effect on people who had preexisting stressors in their lives as a result of their social situations. Namely, we assumed that these individuals would be more likely to experience pandemic-related unemployment and economic distress and therefore be more vulnerable to mental distress.

### 1.3. The Current Study

Although the sustainable development goals formulated by the U.N. [33] were updated following the COVID-19 outbreak, they mainly refer to physical health issues and overlook the important effects of the pandemic on people’s mental health. In keeping with Pearlin’s [30] emphasis on the importance of an individual’s socioeconomic background characteristics in processes of stress, the current study aimed to explore the role of socioeconomic inequities in mental distress during the pandemic among Israeli adults. Thus, we examined (1) the association between various sociodemographic variables, including education, previous illness in the household, and income prior to the coronavirus outbreak and mental distress; (2) the association between unemployment and reduced employment following the COVID-19 outbreak and mental distress; and (3) the association between the economic effects of COVID-19 on individuals and their mental health.

## 2. Materials and Methods

### 2.1. Sample

The sample included 273 participants, between the ages of 20 and 68 (mean age 39.4). The descriptive statistics of the sample are shown in Table 1. Most of the participants were women, Jewish, and born in Israel. More than half were either married or in stable relationships, and about two-third were parents. More than half had a post high school education. Among one-fifth of the participants, either they themselves or a member of their households suffered from one or more illnesses (not including COVID-19). Given that only two participants reported that they or another member of their household had tested positive for COVID-19, this variable was not part of the analysis. Regarding participants’ SES prior to the COVID-19 crisis, almost half of their households earned less than the median income in Israel (8000 NIS), and more than a quarter lived in high-density housing conditions.

The questions regarding the impact of the COVID-19 crisis elicited various negative results: 41% of participant households experienced the unemployment or furlough of at least one provider, or of the need to close their business, and another 16.1% experienced a reduction in the extent of their employment or business activity. In addition, 60.1% experienced an increase in their expenses. More than half experienced a negative change in their economic situation, and more than a tenth took out loans. The mean of the number of items for which financial or instrumental help was needed during the COVID-19 period was 0.61, ranging from zero to six, and included a variety of needs including purchases of food, phones, computers, or laptops; access to the internet; and deliveries of food or medicines. Additionally, 44.3% percent of participants reported that they needed to receive support from family and friends, while 65.6% reported not needing such support.

### 2.2. Process

Data were collected during May 2020, right after the end of the first lockdown in Israel, so as to capture the short-term impact of the crisis. The sample was drawn via two main methods. First, the questionnaire was shared online via the use of an online snowball sampling method. Namely, the researchers shared a link to the questionnaire via WhatsApp and Facebook (Menlo Park, CA, USA), including weekly postings of this link on relevant Facebook groups. Participants were asked to share the link with friends and family. Due to the existence of social distancing rules, using the snowball sampling method was seen as more suitable than the use of other types of non-probability sampling methods. Second, interviewers who are either social workers or social work students went to low-SES neighborhoods and interviewed people in central locations (such as near the supermarket or in playgrounds). The rationale for using this in-person method (while maintaining social distance and mask guidelines) was to locate participants who didn’t use social media due to their age or because of limited access.

### 2.3. Measurement

The development of the questionnaire took place in a four-phase process. First, the researchers collected or adapted questions from previous studies—for example, from the Center for Epidemiological Studies Depression (CES-D) questionnaire [34] regarding mental distress. Second, the questions were carefully examined for ambiguity, wording, and relevance. Third, the content validity of the questions was assessed through discussions held among 11 social workers and social work students in order to check the adaptability and clarity of the questions and to make sure the questions covered the main impact of COVID-19 on the participants. Finally, some of the questions were rephrased according to the feedback that was received.

#### 2.3.1. Mental Distress

Based on the CES-D [34] scale, a seven-item scale including items relevant to the coronavirus period was created by the researchers for the purpose of this study. Participants were asked to indicate how often in the last month they: (1) felt sad or depressed, (2) felt that everything was an effort, (3) experienced sleep problems, (4) had difficulty in concentrating on what they were doing, (5) felt lonely, (6) felt anxious about the financial situation, and (7) felt anxious about their own health and/or the health of their family members. The scale ranged from 1 = all the time to 6 = never. A summed score for these items was calculated. The final answers ranged from seven to 42 points, with a higher score reflecting higher mental distress. Cronbach’s α indicated high internal reliability (0.84).

#### 2.3.2. SES Variables

Monthly household income prior to COVID-19 (1 = less than 3,000 NIS to 5 = 22,000 and above). In addition, questions about the number of people living in the dwelling and the number of rooms in the dwelling were used to calculate housing density.

#### 2.3.3. Changes in Economic Situation following the COVID-19 Outbreak

The participants were asked whether in the aftermath of the COVID-19 outbreak, they or their partner experienced changes in their employment situation (1 = both still employed; 2 = at least one experienced unemployment/furlough; 3 = at least one experienced a reduction in their work hours). In the case of being self-employed (participants or partners) they were asked about changes in their business activity (1 = no change or change for the better; 2 = had to close their business; 3 = business activity has decreased). The answers to these questions were aggregated into one variable: 1 = participants (and/or their partners) are still working, 2 = participants (and/or their partners) are unemployed, on furlough, or had to close their business, or 3 = participants (and/or their partners) experienced a decrease in their work hours either in the context of being employed by someone else or being self-employed). Fifteen (5.4%) participants who had not been engaged in paid work before the COVID-19 outbreak were excluded from the calculation of this variable. In addition, participants were asked whether in the aftermath of the COVID-19 outbreak their household experienced an increase in their expenses, they had a negative change in their economic situation, they took out loans, and/or they received support from family and friends during the crisis.

#### 2.3.4. The Number of Areas in Which They Needed Support

Five possible needs were presented, and the participants were asked to indicate whether they had any of these needs during the crisis. These needs, specifically, were food, toys for the children, computers, access to the internet, and delivery of food and medicines. They could also indicate whether they had other (not previously listed) needs for external support (yes/no).

#### 2.3.5. Sociodemographic Variables

Age, gender, country of birth, marital status, parenthood, education, illness in the family (of the participant or of another member in the household).

### 2.4. Data Analysis

First, bivariate analyses were conducted to assess the associations between the study variables and mental distress as detailed in Table 2. ANOVA and *t*-tests were computed to assess differences in the means of mental distress by categorical variables. Pearson and Spearman’s correlations were employed for continuous explanatory variables. Second, a three-step hierarchical linear regression model was used to estimate mental distress. In the first step, we listed sociodemographic characteristics including income prior to the COVID-19 outbreak (Model 1). In the second step (Model 2), we added the unemployment variables as one main effect of the pandemic, so as to explore their distinct contribution to mental distress. Finally, we included in the third step (Model 3) the various variables of the economic effects of the pandemic, so as to explore their contribution to mental distress above and beyond unemployment. The assumptions of regression analysis of multivariate normality, absence of multicollinearity, and homoscedasticity were met.

### 2.5. Ethical Considerations

The current study adhered to the ethical guidelines of the authors’ university and was approved by the Ethics Committee of the authors’ university. The questionnaire did not include any personally identifying details. The participants signed informed consent either in person or electronically.

## 3. Results

Table 2 includes the bivariate analysis of the associations between the study variables and mental distress. The findings indicated that in terms of sociodemographic variables, whereas age, gender, and being parents were not associated with mental distress, non-married participants (either single, divorced, or widowed) reported higher mental distress. Additionally, participants who had up to a high school education reported higher mental distress than did those with a post high school education. Participants who reported that there were illnesses in the household (apart from COVID-19) also reported higher mental distress. In terms of pre-COVID-19 SES, lower income was related to higher mental distress. Housing density, however, was not related to mental distress.

All of the variables of the economic effects of the pandemic were significantly related to mental distress. People who were either unemployed or experienced a reduction in their work hours reported higher mental distress. People who reported having more expenses, a negative change in their economic situation, and needed to take out loans reported higher mental distress than those who did not. Moreover, people who had a greater number of financial and instrumental support needs during the pandemic and those who had more informal support reported higher mental distress.

Table 3 shows the three-step hierarchical linear regression analysis. The dependent variable was mental distress. According to the first step, participants who were female, with up to a high school education, with a lower income, and with a previous illness in the household had higher mental distress. Participants’ sociodemographic characteristics accounted for 20.5% of the explained variance in mental distress. The second step indicated that unemployment, as well as a reduction in work hours, was positively correlated with mental distress. This step, comprising employment status only, made a distinct contribution of 6% to the total explained variance. The third step, including the various economic effects of COVID-19, showed that needing to take out loans, having a worsened economic situation, and needing financial or instrumental help in a greater number of areas during the crisis positively correlated with mental distress. Receiving support from family and friends was also associated with mental distress. This step made a substantial and distinct contribution to the total explained variance of the model of 23.4%. All three models made a distinct and significant contribution to the explained variance. The entire set of the independent variables accounted for 50.0% of the variance in mental distress, *F* (13,231) = 16.78, *p* < 0.001.

## 4. Discussion

Based on the stress process model [30,31] the aim of this study was to explore the role played by social inequalities in the occurrence of mental distress during the COVID-19 period. The novel coronavirus has been a stressful event for people everywhere as a result of, among other reasons, the health threat it poses to themselves or their loved ones, as well as the economic stress it has caused. Mental health is strongly connected with physical health [14,15]. Therefore, to understand inequalities in physical health and mental health during this pandemic, it is imperative to use a social lens [35].

A social inequality approach to health relates to health disparities “as resulting from the unequal distribution of economic, social and cultural capital” [36] p. 4. According to this approach, the existing data clearly point to social inequalities in terms of the rates of contracting the COVID-19 infection [1,2,3], but this approach is also needed in order to explore the mental health consequences of the pandemic. As such, the current study has identified the sociodemographic factors and economic effects of the pandemic that are related to the mental distress experienced by individuals during this global health crisis.

In this study, we first explored the association between sociodemographic characteristics and mental distress during the pandemic, in keeping with Pearlin’s [30] suggestion that these factors be explored as part of the stress process. The findings strongly indicated that groups who are vulnerable to mental distress in routine times showed the same pattern during the pandemic. Namely, the regression analysis indicated that participants with a low level of education, those who had a lower income prior to COVID-19, and those who had health problems in their households also had higher levels of mental distress. In addition, in line with previous studies, women were also found to have higher levels of mental distress [26,27]. According to these findings, we can assume that groups who were vulnerable before the pandemic and who had already experienced stress due to their social locations, impaired health, and/or economic distress would likely be more vulnerable to experiencing stress in the wake of the pandemic, which might in turn affect their mental health. Lower status groups are also more likely to lack the protective resources to cope with stressors (e.g., social support and financial resources) [37], making them more vulnerable to mental distress [31,32].

Given that unemployment has long been known to be related to mental distress [17,23,38,39], and given that one of the pandemic’s main deleterious effects has been on employment rates [8,40], in this study we examined employment separately from the other effects of the pandemic. Our findings indicated that both being unemployed and experiencing reduced employment were related to higher mental distress. The findings concerning unemployment support studies that have been conducted on the pandemic’s effects [23,41]. The findings of Holingue et al. [26], who examined the impact of the COVID-19 pandemic on mental distress among 5065 adults in the USA in March 2020, indicated that keeping one’s job was a protective factor against mental distress. The importance of employment for mental health was also found in China [28].

However, our findings emphasize that although the strongest effect on mental distress was related to unemployment in the wake of the pandemic, there was another group at risk for impaired mental health: people whose working hours or business activity were reduced as a result of the pandemic. The decline in social status that people may experience as a result of such changes—that is, the need to cut their expenses, the decrease in their standard of living, and the experience of a decline in their perceived social position—might explain this finding. Pratschke et al. [42] identified the relationship between downward social status and the feeling of disenfranchisement, and referred to the connection between objective conditions, in our case reduced working hours, and emotion, in our case mental distress. Similar findings have indicated that an adverse change in social status is related to higher mental distress [43,44]. As such, the outcome of the reduction of working hours, namely the downward social status, might explain the participants’ increased mental distress. Accordingly, Euteneuer and Schäfer [43] stressed the need to promote social interventions to reduce “social pain” resulting from a downward social status. This group may also live in fear of being the next to be furloughed/fired, or the fear that they will have to close their business altogether if the pandemic continues to go on. These fears accompany the experience of decreased income and the other economic effects of the pandemic.

Our findings strongly indicated that various COVID-19 economic effects predicted mental distress above and beyond participants’ sociodemographic factors and employment status. These economic effects, which included needing to take out loans during the crisis, experiencing a worsening economic situation, and having more financial and instrumental needs (in terms of food, electronic devices, food/medicine deliveries, etc.), were the strongest factors in terms of predicting mental distress. These findings support the stress process model: that is, the assumption that vulnerable groups tend to experience co-occurring stressors and that one stressor is very likely to trigger other stressors [30]. Namely, people who are more affected by the crisis are at risk of experiencing more stressors and higher mental distress, if only because unemployment leads to various economic effects which are in turn related to mental distress.

The effect of the economic damages wrought by the pandemic should not be overlooked. Pearlin & Lieberman [45] claimed that poverty can be referred to as a chronic stressor that causes mental distress. The duration of this crisis will likely only deepen and expand the levels of economic distress and poverty around the world [33], even long after the pandemic has passed [46], and therefore its effects on mental health are expected to worsen over time.

The stress process model emphasizes that available resources such as social support can play a protective role as moderators in the stress-distress link [37]. In the current study, we found that participants who received informal support from family and friends were those who reported higher mental distress. One explanation for these findings may be that participants’ networks were not strong enough to provide the support needed to buffer the effects of the crisis on their mental health, either because these networks were weakened in the wake of the pandemic or because they were not that strong even prior to the crisis. Previous studies have in fact indicated the possibility that the social networks of people in low social locations are struggling themselves and would therefore be unable to meet these individuals’ great need for support [47,48], making them even more vulnerable to mental distress. Alternatively, the use of informal support may be associated with a lower sense of dignity and an increase in dependency. Although receiving formal support and medical services are sometimes associated with an erosion in dignity [49,50,51], less has been explored about informal support [50,52]. In terms of our findings, it might be that the need to use informal social support was related to a decreased sense of dignity and an increased sense of dependency, and that both are related to an increase in mental distress. However, this assumption requires further research.

The strengths of the current study and its contribution to the literature derive from its being one of very few studies to explore a wide range of factors relevant to the current crisis and their association with mental distress during the crisis. Along with its merits, however, this study also had a few limitations. First, it was based on a non-representative sample and on an online sampling that does not allow for an assessment of the response rate, thus potentially affecting the generalizability of the findings. This limitation is typical of many studies looking at the effects of the COVID-19 pandemic, as many scholars have had to base their methods on digital questionnaires and interviews, or to limit their sample size [53]. We therefore recommend exploring the current research questions in a representative sample. Second, our data did not include questions concerning previous mental health problems. As such, we could not assess the correlation between this factor and the presence of informal social support or its absence, nor the joint contribution of these factors to the individual’s current mental distress. Lastly, this study took place at the beginning of the crisis; although it provides important data concerning the short-term effects of the pandemic, its ongoing nature and the expanding and deepening economic damages wrought by this pandemic make it incumbent upon professionals in the field to explore the pandemic’s long-term economic effects and the consequent implications for people’s mental health.

The results of this study have several implications for practice. Although it is not surprising that an association was found between certain sociodemographic characteristics and mental distress, the findings do shed light on which groups should be at the focus of interventions in terms of mental health, especially during the pandemic, including women, people with low incomes, and people with medical needs. Additionally, seeing that not only unemployment but also a reduction in work hours posed a threat to mental health should lead policymakers and leaders to consider carefully additional lockdowns as well as to make intensive efforts to revive the employment market. However, given that individuals with low incomes experienced higher rates of job loss and furlough [8], and given that their occupations (mostly in non-standard jobs) were damaged the most, it is to this cohort that different types of concrete and financial support should be given in order to help them both during the crisis and in the years to come. The findings of the current study strengthen the need to see this global health crisis as an economic crisis as well, and to assess the economic effects of this crisis on mental health. This point is especially relevant for people who work in the underground economy.

## 5. Conclusions

Economic inequalities have long been known to affect mental health, but little has been done to address such economic inequalities [54]. Considering the strong relationship that exists between mental health and physical health [15], the current crisis is a wakeup call to assess the needs of the groups most affected economically by the current crisis, so as to protect both their mental health and physical health. Policymakers are therefore called on to use the social model no less than the medical model in treating the ills wrought by COVID-19 [55].

The existing literature has long recognized the association between psychological and social factors and mental distress. However, in terms of the current economic crisis and the inequality in the pandemic’s effects, people’s sociodemographic data and pre- and post-pandemic SES should be looked at as the main factors that can potentially affect mental health. Accordingly, they must be taken into consideration in any social intervention. Additionally, the pandemic has resulted in an economic crisis and distress, and studies have pointed to an association between economic distress, stress and mental distress, as well as an association between mental distress, illness, and mortality. As such, it seems that exploring the economic factors that are related to mental distress, especially during this once-in-a-century pandemic, may help stop this vicious cycle.

## Figures and Tables

**Table 1:** Descriptive statistics of the study variables (*N* = 273).

Variables	*N*	*%/Mean (SD)*
**Sociodemographic**		
Age		
20–24	29	10.6
25–35	90	33
36–54	119	43.6
55–68	35	12.8
Female (=1)	187	68.5%
Immigrant (=1)	44	16.3%
Married (=1)	168	61.5%
Children (=1)	179	65.6%
Education (post high school =1)	146	53.5%
Health problem in the household (=1)	60	20.0%
**SES** Monthly household income		
Less than 3000 NIS	26	9.5%
3000–7000 NIS	94	36.3%
7000–15,000 NIS	64	23.4%
15,000–22,000 NIS	49	17.9%
22,000 and above	34	12.5%
Housing density * (=1)		27.5%
**Economic effects of COVID-19**		
Employment		
Employed	94	34.4%
Reduced employment	44	16.1%
Unemployed **	112	41.0%
Increase in expenses (=1)	164	60.1%
Negative change in economic situation (=1)	150	54.9%
Took out loans (=1)	32	11.7%
Needs for support (0–6)		0.61 (1.03)
Received informal support (=1)	121	44.3%
Mental Distress (7–42)		20.34 (8.47)

* Housing density computed according to the index of PPR-Person Per Room; ** Note that being unemployed refers to those who had been working before the coronavirus outbreak (either as salaried workers or as self-employed individuals) and who had been furloughed or fired, as well as self-employed people whose businesses had closed.

**Table 2:** Bivariate analysis of mental distress by the study variables (*N* = 273).

Sociodemographic	Mental Distress Mean (SD)	*F*/*t*-Test/Pearson Correlation
**Age**		
20–24	22.52 (8.81)	*F* = 1.56
25–35	19.18 (7.31)	
36–54	20.25 (8.85)	
55–68	22.09 (9.49)	
**Gender**		
Male	19.38 (8.92)	*t* = 1.25
Female	20.80 (8.23)	
**Birth country**		
Israel	19.83 (8.15)	*t* = 3.09 **
Other	24.02 (9.23)	
**Marital status**		
Married	19.21 (7.90)	*t* = 2.67 **
Other	22.07 (9.04)	
**Have kids**		
Yes	20.23 (8.49)	*t* = 0.30
No	20.56 (8.48)	
**Education**		
High school or below	22.96 (9.01)	*t* = 4.79 ***
Post high school	17.99 (7.29)	
**Health problem in the household**		
Yes	23.79 (8.51)	*t* = 5.72 ***
No	17.97 (7.60)	
***SES before COVID-19***		
**Income**		*r* = −0.33 ***
**Housing density**		
Yes	20.51 (8.34)	*t* = 0.41
No	20.02 (8.49)	
***Economic effects of COVID-19***		
**Employment**		
Continued	16.17 (6.40)	*F*= 17.57 ***
Unemployed	20.90 (8.09) ^a^	
Reduced employment	22.70 (8.50) ^a^	
**Increase in expenses**		
Yes	23.79 (8.52)	*t* = 5.72 ***
No	17.97 (7.60)	
**Loans**		
Yes	26.57 (8.58)	*t* = 4.44 ***
No	19.51 (8.12)	
**Worsened economic situation**		
Yes	23.64 (8.58)	*t* = 8.69 ***
No	15.79 (5.80)	
**Needs for support (0–6)**		r = 0.42 ***
**Received informal support**		
Yes	23.91 (8.21)	*t* = 6.85 ***
No	17.17 (7.38)	

** *p* ≤ 0.01, *** *p* ≤ 0.001; ^a^ Significantly different (*p* < 0.001) from these who were still employed.

**Table 3:** Hierarchical multiple regression analysis for predicting mental distress during COVID-19 (*N* = 273).

Variables	Model 1			Model 2			Model 3		
	B	S.E.B	β	B	S.E.B	β	B	S.E.B	β
**Sociodemographic**									
Gender (female = 1)	2.68	1.07	0.15	2.11	1.05	0.12	1.09	0.09	0.06
Country of birth (immigrant = 1)	2.24	1.38	0.10	2.07	1.34	0.09	1.92	1.13	0.09
Marital status (married = 1)	0.18	1.13	0.01	−0.56	1.11	−0.03	−0.73	0.93	−0.04
Education (post high school = 1)	−2.75	1.23	−0.17 *	−2.27	1.20	−0.14	−1.20	1.01	−0.07
Income (1–5)	−1.48	0.56	−0.20 **	−0.95	0.56	−0.13	−0.07	0.48	−0.01
Health problems (yes = 1)	4.43	1.25	0.21 ***	4.24	1.21	0.20 ***	3.68	1.01	0.18 ***
**Employment**									
Unemployed (=1)				4.58	1.13	0.28 ***	0.88	1.10	0.05
Reduced employment (=1)				3.89	1.43	0.17 **	2.29	1.32	0.10
**Economic effects of COVID-19**									
Increase in expenses (yes = 1)							−0.99	0.94	−0.06
Loans (yes = 1)							3.05	1.32	0.12 *
Worsened economic situation (yes = 1)							3.62	1.08	0.22 ***
Needs for support (0–6)							1.94	0.43	0.25 ***
Received informal support (yes = 1)							4.39	0.88	0.26 ***
*R* ^2^	0.21 ***			0.26 ***			0.50 ***		
Δ*R*^2^	-			0.06 ***			0.24 ***		

* *p* ≤ 0.05, ** *p* ≤ 0.01, *** *p* ≤ 0.001.

## Data Availability

The data that support the findings of this study are available from the corresponding author upon reasonable request.

## References

[1] Adhikari S., Pantaleo N.P., Feldman J.M., Ogedegbe O., Thorpe L., Troxel A.B. (2020). Assessment of Community-Level Disparities in Coronavirus Disease 2019 (COVID-19) Infections and Deaths in Large US Metropolitan Areas. JAMA Netw. Open.

[2] Guan W., Liang W., Zhao Y., Liang H., Chen Z., Li Y., Liu X.Q., Chen R.C., Tang C.L., Wang T. (2020). Comorbidity and its impact on 1590 patients with COVID-19 in China: A nationwide analysis. Eur. Respir. J..

[3] Rentsch C.T., Kidwai-Khan F., Tate J.P., Park L.S., King J.T., Skanderson M., Hauser R.G., Schultze A., Jarvis C.I., Holodniy M. (2020). Patterns of COVID-19 testing and mortality by race and ethnicity among United States veterans: A nationwide cohort study. PLoS Med..

[4] OECD (2021). Risks that Matter 2020: The Long Reach of COVID-19.

[5] Rapid Perception Survey on COVID19 Awareness and Economic Impact. https://reliefweb.int/sites/reliefweb.int/files/resources/Perception-Survey-Covid19.pdf.

[6] Nicola M., Alsafi Z., Sohrabi C., Kerwan A., Al-Jabir A., Iosifidis C., Agha M., Agha R. (2020). The socio-economic implications of the coronavirus pandemic (COVID-19): A review. Int. J. Surg..

[7] Martin A., Markhvida M., Hallegatte S., Walsh B. (2020). Socio-Economic Impacts of COVID-19 on Household Consumption and Poverty. Econ. Disasters Clim. Chang..

[8] Hardship is Greatest among Vulnerable Israelis already Struggling Financially. https://socialpolicyinstitute.wustl.edu/hardship-is-greatest-among-vulnerable-israelis-already-struggling-financially/.

[9] ILO Monitor COVID-19 and the World of Work. https://www.oitcinterfor.org/en/node/7770.

[10] Record Rise in OECD Unemployment Rate in April 2020. http://www.oecd.org/sdd/labour-stats/unemployment-rates-oecd-06-2020.pdf.

[11] World Health Organization (2013). Transforming and Scaling Up Health Professionals’ Education and Training: World Health Organization Guidelines 2013.

[12] Goldberg D.P., Huxley P. (1992). Common Mental Disorders: A Bio-Social Model.

[13] Bao Giang K., Viet Dzung T., Kullgren G., Allebeck P. (2010). Prevalence of mental distress and use of health services in a rural district in Vietnam. Glob. Health Action.

[14] Ahmad F., Jhajj A.K., Stewart D.E., Burghardt M., Bierman A.S. (2014). Single item measures of self-rated mental health: A scoping review. BMC Health Serv. Res..

[15] Yang L., Zhao M., Magnussen C.G., Veeranki S.P., Xi B. (2020). Psychological distress and mortality among US adults: Prospective cohort study of 330 367 individuals. J. Epidemiol. Community Health.

[16] Darin-Mattsson A., Andel R., Celeste R.K., Kåreholt I. (2018). Linking financial hardship throughout the life-course with psychological distress in old age: Sensitive period, accumulation of risks, and chain of risks hypotheses. Soc. Sci..

[17] Jefferis B.J., Nazareth I., Marston L., Moreno-Kustner B., Bellón J.Á., Svab I., Rotar D., Geerlings M.I., Xavier M., Goncalves-Pereira M. (2011). Associations between unemployment and major depressive disorder: Evidence from an international, prospective study (the predict cohort). Soc. Sci..

[18] Mossakowski K.N. (2015). Disadvantaged family background and depression among young adults in the United States: The roles of chronic stress and self-esteem. Stress Health.

[19] Valk I. (2017). Social Capital as a Solution to Mental Health Problems: The Effect of Social Capital On ‘Depression’ and ‘Loneliness’ Via the Effect of Social Cohesion and Civic Engagement. Master’s Thesis.

[20] Ward E., Mengesha M. (2013). Depression in African American men: A review of what we know and where we need to go from here. Am. J. Orthopsychiatry.

[21] Wu Q., Xie B., Chou C.P., Palmer P.H., Gallaher P.E., Anderson Johnson C. (2010). Understanding the effect of social capital on the depression of urban Chinese adolescents: An integrative framework. Am. J. Community Psychol..

[22] Zhang Y., Jiang J. (2019). Social capital and health in China: Evidence from the Chinese general social survey 2010. Soc. Indic. Res..

[23] Achdut N., Refaeli T. (2020). Unemployment and psychological distress among young people during the COVID-19 pandemic: Psychological resources and risk factors. Int. J. Environ..

[24] Mushonga D.R., Henneberger A.K. (2019). Protective factors associated with positive mental health in traditional and nontraditional black students. Am. J. Orthopsychiatry.

[25] Canady V.A. (2020). APA poll finds rise in anxiety related to COVID-19, election. Ment. Health Wkly..

[26] Holingue C., Kalb L.G., Riehm K.E., Bennett D., Kapteyn A., Veldhuis C.B., Jhonson R.M., Fallin D., Kreuter F., Stuart E.A. (2020). Mental Distress in the United States at the Beginning of the COVID-19 Pandemic. Am. J..

[27] Rens E., Smith P., Nicaise P., Lorant V., Van den Broeck K. (2021). Mental distress and its contributing factors among young people during the first wave of COVID-19: A Belgian survey study. Front. Psychiatry.

[28] Shi L., Lu Z.A., Que J.Y., Huang X.L., Liu L., Ran M.S., Gong M.Y., Yuan K., Yan W., Sun Y. (2020). Prevalence of and risk factors associated with mental health symptoms among the general population in China during the coronavirus disease 2019 pandemic. JAMA Netw. Open.

[29] Pearlin L.I. (1981). The stress process. J. Health Soc. Behav..

[30] Pearlin L.I. (1989). The sociological study of stress. J. Health Soc. Behav..

[31] Pearlin L.I., Schieman S., Fazio E.M., Meersman S.C. (2005). Stress, health, and the life course: Some conceptual perspectives. J. Health Soc. Behav..

[32] Thoits P.A. (2010). Stress and health: Major findings and policy implications. J. Health Soc. Behav..

[33] The Sustainable Development Goals: Our Framework for COVID-19 Recovery. https://www.un.org/sustainabledevelopment/sdgs-framework-for-covid-19-recovery/.

[34] Andresen E.M., Malmgren J.A., Carter W.B., Patrick D.L. (1994). Screening for depression in well older adults: Evaluation of a short form of the CES-D. Am. J. Prev. Med..

[35] Tai D.B.G., Shah A., Doubeni C.A., Sia I.G., Wieland M.L. (2021). The Disproportionate Impact of COVID-19 on Racial and Ethnic Minorities in the United States. Clin. Infect. Dis..

[36] Abel T. (2008). Cultural capital and social inequality in health. J. Epidemiol. Community Health.

[37] Pearlin L.I. (2010). The life course and the stress process: Some conceptual comparisons. J Gerontol. B Psychol. Sci. Soc. Sci..

[38] Pompili M., Innamorati M., Sampogna G., Albert U., Carmassi C., Carrà G., Cirulli F., Erbuto D., Luciano M., Giulia Nanni M. (2022). The impact of COVID-19 on unemployment across Italy: Consequences for those affected by psychiatric conditions. J. Affect. Disord..

[39] Reneflot A., Evensen M. (2014). Unemployment and psychological distress among young adults in the Nordic countries: A review of the literature. Int. J. Soc. Welf..

[40] Bauer A., Weber E. (2021). COVID-19: How much unemployment was caused by the shutdown in Germany?. Appl. Econ..

[41] Gualano M.R., Lo Moro G., Voglino G., Bert F., Siliquini R. (2020). Effects of COVID-19 lockdown on mental health and sleep disturbances in Italy. Int. J. Environ..

[42] Pratschke J., Vitale T., Morelli N., Cousin B., Piolatto M., Del Fabbro M. (2021). Electoral support for the 5 Star Movement in Milan: An ecological analysis of social and spatial factors. J. Urban Aff..

[43] Euteneuer F., Schäfer S.J. (2018). Brief report: Subjective social mobility and depressive symptoms in Syrian refugees to Germany. J Immigr. Minor Health.

[44] Nicklett E.J., Burgard S.A. (2009). Downward social mobility and major depressive episodes among Latino and Asian-American immigrants to the United States. Am. J. Epidemiol..

[45] Pearlin L.I., Lieberman M.A. (1979). Social sources of emotional distress. Community Ment. Health J..

[46] Hagger M.S., Keech J.J., Hamilton K. (2020). Managing stress during the coronavirus disease 2019 pandemic and beyond: Reappraisal and mindset approaches. Stress Health.

[47] Desmond M. (2012). Disposable Ties and the Urban Poor. Am. J. Sociol..

[48] Refaeli T., Achdut N. (2020). Perceived poverty, perceived income adequacy and loneliness in Israeli young adults: Are social capital and neighbourhood capital resilience factors?. Health Soc. Care Community.

[49] Jacobson N. (2009). Dignity violation in health care. Qual. Health Res..

[50] Persson C., Benzein E., Morberg Jämterud S. (2020). Dignity as an intersubjective phenomenon: Experiences of dyads living with serious illness. Qual. Health Res..

[51] Wright G., Neves D., Ntshongwana P., Noble M. (2015). Social assistance and dignity: South African women’s experiences of the child support grant. Dev. S. Afr..

[52] Dunér A., Nordström M. (2007). The roles and functions of the informal support networks of older people who receive formal support: A Swedish qualitative study. Ageing Soc..

[53] Bath Social and Development Research Ltd. Qualitative Research in a Pandemic. https://bathsdr.org/qualitative-research-in-a-pandemic.

[54] Gil-Rivas V., Handrup C.T., Tanner E., Walker D.K. (2019). Global Mental Health: A Call to Action. Am. J. Orthopsychiatry.

[55] Patel J.A., Nielsen F.B.H., Badiani A.A., Assi S., Unadkat V.A., Patel B., Ravindrane R., Wardle H. (2020). Poverty, inequality and COVID-19: The forgotten vulnerable. Public Health.

